# The Chemopotential Effect of *Annona muricata* Leaves against Azoxymethane-Induced Colonic Aberrant Crypt Foci in Rats and the Apoptotic Effect of Acetogenin Annomuricin E in HT-29 Cells: A Bioassay-Guided Approach

**DOI:** 10.1371/journal.pone.0122288

**Published:** 2015-04-10

**Authors:** Soheil Zorofchian Moghadamtousi, Elham Rouhollahi, Hamed Karimian, Mehran Fadaeinasab, Mohammad Firoozinia, Mahmood Ameen Abdulla, Habsah Abdul Kadir

**Affiliations:** 1 Biomolecular Research Group, Biochemistry Program, Institute of Biological Sciences, Faculty of Science, University of Malaya, Kuala Lumpur, Malaysia; 2 Department of Biomedical Science, Faculty of Medicine, University of Malaya, Kuala Lumpur, Malaysia; 3 Department of chemistry, Faculty of Science, University of Malaya, Kuala Lumpur, Malaysia; Indian Institute of Technology, INDIA

## Abstract

*Annona muricata* has been used in folk medicine for the treatment of cancer and tumors. This study evaluated the chemopreventive properties of an ethyl acetate extract of *A*. *muricata* leaves (EEAML) on azoxymethane-induced colonic aberrant crypt foci (ACF) in rats. Moreover, the cytotoxic compound of EEAML (Annomuricin E) was isolated, and its apoptosis-inducing effect was investigated against HT-29 colon cancer cell line using a bioassay-guided approach. This experiment was performed on five groups of rats: negative control, cancer control, EEAML (250 mg/kg), EEAML (500 mg/kg) and positive control (5-fluorouracil). Methylene blue staining of colorectal specimens showed that application of EEAML at both doses significantly reduced the colonic ACF formation compared with the cancer control group. Immunohistochemistry analysis showed the down-regulation of PCNA and Bcl-2 proteins and the up-regulation of Bax protein after administration of EEAML compared with the cancer control group. In addition, an increase in the levels of enzymatic antioxidants and a decrease in the malondialdehyde level of the colon tissue homogenates were observed, suggesting the suppression of lipid peroxidation. Annomuricin E inhibited the growth of HT-29 cells with an IC50 value of 1.62 ± 0.24 μg/ml after 48 h. The cytotoxic effect of annomuricin E was further substantiated by G1 cell cycle arrest and early apoptosis induction in HT-29 cells. Annomuricin E triggered mitochondria-initiated events, including the dissipation of the mitochondrial membrane potential and the leakage of cytochrome *c* from the mitochondria. Prior to these events, annomuricin E activated caspase 3/7 and caspase 9. Upstream, annomuricin E induced a time-dependent upregulation of Bax and downregulation of Bcl-2 at the mRNA and protein levels. In conclusion, these findings substantiate the usage of *A*. *muricata* leaves in ethnomedicine against cancer and highlight annomuricin E as one of the contributing compounds in the anticancer activity of *A*. *muricata* leaves.

## Introduction

The complex and multistep process of carcinogenesis generally involves three main stages: initiation, promotion and progression [1]. Perturbations in the genetic level as a result of exposure to carcinogenic agents, including chemical, physical or viral agents, can trigger the initiation phase [2]. Morphological changes and the expansion of altered cells are paramount characterizations of the promotion stage. In the progression stage, genotypic and phenotypic conversions are accompanied with malignancy and metastasis [3].

Colorectal cancer evolves through the deregulation and aberrant growth of epithelial cells in the appendix, colon or rectum [4]. Early detection is pivotal to reduce the number of colorectal cancer victims [5]. The promotion stage in this type of cancer is characterized by aberrant crypt foci (ACF), which are the earliest identifiable precancerous lesions in colon carcinogenetic models in both animals and humans [6]. Therefore, monitoring for ACF is widely employed to inspect the effects of various anticarcinogens against colorectal cancer [7]. The carcinogen azoxymethane (AOM, C_2_H_6_N_2_O), an oxide of azomethane, has been widely utilized to start the initiation phase of colorectal cancer, thus stimulating AOM-induced ACF in experimental models. This carcinogenic agent is particularly effective for the induction of colorectal cancer [8].

The evasion of apoptosis is an important property of human cancers, which effectively cause tumor formation and cancer progression [9]. The resistance of cancer cells to apoptosis in response to pertinent stimuli is a critical rationale behind treatment failure [10,11]. Therefore, the majority of strategies used in cancer treatment, including chemotherapy and radiation therapy, are generally based on inducing apoptosis in cancer cells [12]. The induction of apoptosis in cancer cells is primarily triggered through two apoptosis pathways: the intrinsic (mitochondrial) pathway and the extrinsic (receptor) pathway, which both eventually lead to the executioner phase via caspase activation [13]. Caspases, including initiators and executioners, are a family of enzymes that act as death effector proteins in different types of cell death [14].

The long history of employing natural products in ethnomedicine with low-prices and limited side effects, in contrast to expensive synthetic drugs with severe adverse side effects, was the main reason for the development of new pharmaceutical drugs from natural sources [15,16]. In addition, a marked similarity between numerous plant ingredients and the compositions of the human body has evolved acceptable immunity to the majority of plant-derived products. Over the past few decades, natural compounds with apoptosis-inducing effects have attracted noteworthy interest in the area of anticancer pharmaceutical agents [15,16]. There is a growing trend towards natural products with high hopes for new anticancer drugs with similar effect to camptothecin (*Camptotheca acuminata*) and paclitaxel (*Taxus brevifolia*) [17]. Numerous plants were subjected to detailed scientific scrutiny and plenty of them, including *Allium sativum* [18], *Andrographis paniculata* [19], *Glycine max* [20], *Gynura procumbens* [21], *Panax ginseng* [22], *Zingiber officinale* [23], reported to possess noteworthy anticancer and antitumor activity. Therefore, screening for new plant-derived anticancer agents may lead to cost-effective chemotherapeutic drugs with diminished side effects while maintaining therapeutic efficacy.

*Annona muricata* L. (*A*. *muricata*), commonly named “graviola” or “soursop”, is a small tropical tree from the Annonaceae family, also known the custard apple family [24,25]. This popular fruit tree, known as “the cancer killer”, has an extensive traditional history in the treatment of cancer and tumors in South America and tropical Africa, especially Nigeria [26–28]. Different studies on *A*. *muricata* leaves have demonstrated noteworthy cytotoxic effects against various cancer cell lines [28–30]. In our previous cytotoxicity screening, the ethyl acetate extract of *A*. *muricata* leaves (EEAML) was found to induce apoptosis in A549, HT-29 and HCT-116 cancer cells [28,30]. Moreover, the safety of EEAML for animal studies was proven by the acute toxicity study in rats, which showed no sign of toxicity, even at a high dose of 2 g/kg [25]. The present study was designed to evaluate the chemopreventive properties of EEAML on the development and growth of AOM-induced colorectal cancer in rats by analyzing the incidence of ACF. Moreover, EEAML was subjected to a bioassay-guided approach to isolate the cytotoxic compound annomuricin E from *A*. *muricata* and examine its apoptosis-inducing effects.

## Materials and Methods

### General Experimental Procedures

Column chromatography (CC) was run on a silica gel 60 column (40–63 μm particle size, Merck, Darmstadt, Germany). Thin layer chromatography (TLC) was performed on an aluminum supported silica gel 60 F_254_ column (Merck). Preparative TLC (PTLC) was run on glass coated with silica gel 60 F_254_ (Merck). ^1^H NMR and ^13^C NMR spectra were analyzed in CDCl_3_ on a JEOL JNM-FX500 spectrometer (Tokyo, Japan). The ultraviolet absorption spectra were obtained on a Shimadzu UV-160A spectrophotometer (Kyoto, Japan) using methanol (CH_3_OH) as a solvent. The separation was performed on a HPLC machine (Gilson, Inc., Middleton, WI, USA) with a photodiode array (PDA) detector and an ODS C_18_ column (Phenomenex, Torrance, CA, USA). The mass spectra were measured with an Agilent 6530 mass spectrometer (Santa Clara, CA, USA). The infrared spectra were obtained on a Perkin Elmer Spectrum 400-FTIR spectrometer (Waltham, MA, USA) with CHCl_3_ as a solvent.

### Plant Material and Extraction

Fresh leaves of the *A*. *muricata* plant were collected from Ipoh, Malaysia, in March 2013. We obtained prior permission from all landowners and no endangered or protected species were sampled. Botanical identification was performed by Dr. Yong Kien Thai, an ethnobotanist from the Department of Biological Sciences at the University of Malaya. A voucher specimen (No. KLU47978) has been deposited in the herbarium of the University of Malaya. The dried powdered leaves (3 kg) of *A*. *muricata* were macerated with ethyl acetate (3 × 2,500 ml) three times at room temperature. The extracting solvent was decanted and concentrated to dryness using a rotary vacuum evaporator (Buchi Labortechnik AG, Flawil, Switzerland) at 40°C. The percentage yield after extraction was 3.9% (117 g). The isolated extract was dissolved in 10% Tween-20 (Sigma, St. Louis, MO, USA) to prepare 250 mg/kg and 500 mg/kg stocks for further experiments.

### Animals and Ethics Statement

Healthy adult male *Sprague Dawley* rats (180–250 g weight) were provided by the Animal House of the AEU (Animal Experimental Unit, University of Malaya) in clean, sterile and polyvinyl cages. Rats were housed in a standard animal room air-conditioned at 22–24°C and 55% humidity with a normal pellet diet and water *ad libitum*. Light and dark cycles were scheduled for 12 h each. At the end of the experiment, each animal was sacrificed under ketamine/xylazine anesthesia. The animal studies were performed in the AEU after approval of the protocol by the FOM Institutional Animal Care and Use Committee, University of Malaya (FOM IACUC, ethic No.: 2014-03-05-PHAR/R/SZM). All rats received humane care in accordance with national guidelines (Guide for the Care and Use of Laboratory Animals) [31].

### Experimental Protocols

The experiment was performed as previously described in detail [32]. Thirty male rats (n = 6 per group) in five groups (negative control, cancer control, low dose of EEAML, high dose of EEAML and treatment control) were subcutaneously injected once a week for two consecutive weeks according to the “Induction” column in Table 1. Then, all of the rodents were orally fed once a day for two months based on the experimental design (Table 1), except for the treatment control group, which was intra-peritoneally injected with 35 mg/kg of 5-FU (Sigma, St. Louis, MO, USA) for five consecutive days. The condition of the animals was observed every morning throughout the experimental period.

**Table 1:** The experimental design and specifications.

**Group**	**Description**	**Induction**	**Treatment**
**A**	Negative control	normal saline (15 mL/kg)	10% Tween-20 (5 ml/kg)
**B**	Cancer control	AOM (15 mL/kg)	10% Tween-20 (5 ml/kg)
**C**	Low dose	AOM (15 mL/kg)	EEAML (250 mg/kg)
**D**	High dose	AOM (15 mL/kg)	EEAML (500 mg/kg)
**E**	Treatment control	AOM (15 mL/kg)	5-FU (35 mg/kg)

### Counting the ACF

To determine the intensity of colonic ACF formation after 10 weeks of injection with AOM, ACF counting was performed as previously described in detail [33]. In brief, rats were anesthetized with a high dose of ketamine (30 mg/kg, 100 mg/mL) and xylazine (3 mg/kg, 100 mg/mL) under aseptic conditions. The excised colon was flushed with phosphate buffered saline (PBS, Sigma), opened longitudinally and fixed flat between filter papers overnight at 4°C using 10% buffered formalin. Equal lengths of the proximal and distal portions of the fixed colons were stained with 0.5% methylene blue solution. After washing away the excess stain, topographic analysis was performed under a light microscope (Nikon, Tokyo, Japan) to score the total number of ACF, as well as the number of crypts per focus.

### Immunohistochemistry

Immunohistochemical evaluation of proliferating cell nuclear antigen (PCNA), Bax and Bcl-2 proteins was performed on deparaffinized tissue sections using the commercial Dako ARK Peroxidase kit (DAKO, Carpinteria, CA, USA) according to the vendor’s instructions. In brief, the antigen retrieval process of tissue sections was performed using 10 mM citrate buffer (pH 6.0); the tissue sections were then washed with PBS and blocked with peroxidase blocking buffer. Next, the tissue sections were incubated with diluted mouse PCNA (1:100, Cat: ab2426), Bax (1:100, Cat: ab7977) and Bcl-2 (1:100, Cat: ab7973) antibodies (Abcam, Cambridge, MA, USA) for 15 min. All of the slides were then incubated with the appropriate amount of streptavidin-HRP for 30 min at room temperature. The slides were developed with a diaminobenzidine (DAB) substrate-chromogen system and were counterstained in hematoxylin. The measurement of the PCNA labeling index (PI) was calculated using the formula below [34].

$$PI\;=\;\frac{number\;of\;positive\;cells}{total\;number\;of\;epithelial\;cells}\;\times 100$$

### Enzymatic Antioxidants

The colon tissue samples were homogenized in phosphate buffer solution (10% w/v) using a Teflon homogenizer (Polytron, Heidolph RZR 1, Germany). The supernatant was separated after centrifugation at 4000 rpm for 10 min at -4°C. The antioxidant enzymatic activities were assessed using catalase (CAT), glutathione peroxidase (GPx) and superoxide dismutase (SOD) assay kits (Cayman Chemical, Ann Arbor, MI, USA) following the vendor’s instructions.

### Malondialdehyde

A commercial kit (Cayman Chemical, Ann Arbor, MI, USA) was used to measure the malondialdehyde (MDA) levels in colon tissue homogenates employing the thiobarbituric acid reactive substances (TBARS) assay as previously described in detail [35]. The TBARS assay determines the MDA level, which represents the intensity of lipid peroxidation.

### Bioassay-Guided Fractionation and Isolation of Compound

**Fig 1:**
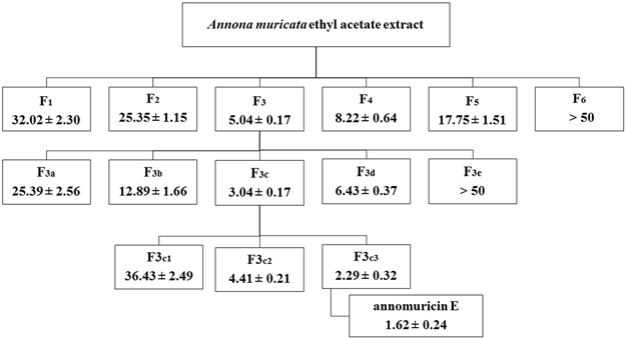
Schematic representation of the bioassay-guided isolation of annomuricin E from EEAML. The cytotoxic effect of each fraction was examined against HT-29 cells for 48 h using an MTT assay. The IC_50_ values (μg/ml) represent the means ± SEM of three independent experiments.

Based on the results of the MTT assay from our previous study [28], the ethyl acetate extract of *A*. *muricata* leaves was selected for further purification. The crude ethyl acetate extract (9 g) was subjected to CC, which was performed on a silica gel 60 column. The column was eluted with hexane/ethyl acetate mixtures of increasing polarity (70:30 → 0:100). TLC analysis was performed on the collected eluates, and those samples displaying similar R_f_ values on the TLC were pooled to yield six fractions (designated F_1_-F_6_). Each fraction was subjected to an MTT assay. Because fraction 3 (F_3_) elicited the strongest cytotoxic effect on HT-29 cells (Fig 1), it was used for further purification. Approximately 3.9 g of the bioactive fraction was subjected to another step of chromatography on a silica 60 micro column and were eluted stepwise with ethyl acetate/dichloromethane mixtures of increasing polarity (70:30 → 0:100), and five fractions (F_3a_-F_3e_) were obtained. The bioactive fraction F_3c_ (1.8 g) was further fractionated on a preparative TLC using dichloromethane/methanol mixtures of increasing polarity (70:30). Another three fractions (F3_c1_-F3_c3_) were obtained and subjected to an MTT assay. The successive separation of F3_c3_ (0.75 g) by preparative HPLC with an ODS C18 column (4.6 x 250 mm, 5.0 μm, 70 A) and the mobile system (50–100% MeOH-H2O ingredient, detection at 220 nm, 7 ml/min) yielded 5 mg of annomuricin E (0.0025%).

### Annomuricin E

Oil, [α]D +13 (c = 0.5 MeOH) UV (MeOH) _λmax_, 220 nm; IR (CHCl_3_) _νmax_ 3401, 1704 cm^-1^; ^1^H NMR (CDCl_3_, 500 MHz), ^13^C NMR (CDCl_3_, 125 MHz) (see Table 2); LCMS m/z 613.4734 [M+1]^+^—(calculated for C_35_H_64_O_8_).

**Table 2:** ^1^H NMR (500 MHz) and ^13^C NMR (125 MHz) spectral data of annomuricin E in CDCl_3_ (δ in ppm, *J* in Hz).

**Position**	^1^ **H-NMR (δ ppm)**	^13^ **C-NMR (δ ppm)**
1		174.7
2		131.2
3	2.49 *m*	33.4
4	3.83 *m*	70.0
5	1.43 *m*	37.3
6–8	1.46 *m*	22.7
9	1.40 *m*	32.0
10	3.40 *m*	77.1
11	3.41 *m*	75.2
12–14	1.99 *m*	32.0
15	3.77 *m*	75.2
16	3.83 *m*	81.7
17–18	2.40 *m*	29.4
19	3.83 *m*	79.5
20	3.77 *m*	75.2
21–31	1.28	29.5
32	0.85 *m*	19.1
33	7.17 *d* (2.2)	152.0
34	5.04 *m*	78.1
35	1.40 *d* (2.1)	14.2

### Cell Culture

CCD841 (normal human colon epithelial cells) and HT-29 (human colon cancer cells) were purchased from the American Type Cell Collection (ATCC, Manassas, VA, USA). The cells were maintained at 37°C in a humidified atmosphere of 5% CO_2_. The culture medium consisted of Dulbecco’s Modified Eagles medium (Sigma) that was supplemented with 10% Fetal Bovine Serum (PAA Laboratories, Pasching, Australia), 100 μg/ml streptomycin (Sigma) and 100 U/ml penicillin (Sigma). The untreated medium containing 0.1% vehicle DMSO was applied as the negative control for all of the assays in the *in vitro* study.

### MTT Assay

Cell viability analysis was performed using the MTT assay as described previously [36]. In brief, cells (5 × 10^4^ cells/ml) at the exponential phase of growth were seeded in a 96-well plate and treated with serial concentrations of the tested agent (0.62, 1.25, 2.5, 5, 10, 20, 40 and 80 μg/ml) for 12, 24 and 48 h. 5-FU, a standard anticancer drug, was used as a positive control in this assay. After incubation, 20 μl of the MTT solution (5.0 mg/ml, Sigma) was loaded into each well, and the cells were further incubated at 37°C for 4 h. DMSO (150 μl) was then used to dissolve the formazan crystals. The cytotoxicity against cancer and normal cells was measured at the absorbance of 570 nm using an ELISA reader (Asys UVM340, Eugendorf, Austria). The data were then processed, and the antiproliferative potential of the tested agents was expressed as IC_50_ values, the concentration that causes a 50% inhibition of cell growth.

### Lactate Dehydrogenase (LDH) Release Assay

To further confirm the cytotoxic effects of annomuricin E on HT-29 cells, the LDH release assay was performed using the Pierce LDH Cytotoxicity Assay Kit (Thermo Scientific, Pittsburgh, PA, USA) as previously described [28]. Briefly, HT-29 cells at the exponential phase of growth were treated with different concentrations of annomuricin E and Triton X-100 (positive control) for 24 h. After the incubation, the treated HT-29 cells were exposed to the LDH reaction solution (100 μl) for 30 min. The red color intensity, representing the level of released LDH, was then measured at 490 nm using the Tecan Infinite 200 Pro (Tecan, Männedorf, Switzerland) microplate reader. The result of LDH release was calculated as a percentage of the positive control.

### Cell Cycle Assay

To determine the effect of annomuricin E on the cell cycle distribution, flow cytometric analysis was performed as described previously [37]. In brief, HT-29 cells (1 × 10^6^ cells/ml) at the exponential phase of growth were seeded in 6-well plates and treated with annomuricin E at the IC_50_ concentration for 12, 24 and 48 h. After incubation, the treated HT-29 cells were harvested, washed twice with ice-cold PBS and fixed overnight at 4°C with 90% ethanol. The following day, the cells were washed and stained with propidium iodide (PI, 100 μl, 1 mg/ml). The cellular RNA was degraded using the enzyme RNAse A (200 μg/ml, Sigma). The stained cells were instantly examined using a BD FACSCanto II flow cytometer (BD Biosciences, San Jose, CA, USA) by analyzing 10,000 cells per sample. The data were processed using ModFit LT software (Verity Software House, Inc., Topsham, ME, USA).

### Quantitative Detection of Early and Late Apoptosis

Flow cytometric analysis was performed to quantify early and late apoptosis in treated HT-29 cells using the commercial BD Pharmingen Annexin V-FITC Apoptosis Detection kit (APOAlert Annexin V; Clontech, Mountain View, CA, USA). Briefly, HT-29 cells (1 × 10^5^ cells/ml) at the exponential phase of growth were incubated with annomuricin E at the IC_50_ concentration for 12, 24 and 48 h. After incubation, the treated cells were harvested, washed twice with PBS and suspended in the Annexin-V binding buffer. The cells were then supplemented with Annexin-V-FITC and PI, according to the vendor’s instructions. The stained cells were examined using a BD FACSCanto II flow cytometer. Early and late apoptotic cells and necrotic cells were quantitatively detected using a quadrant statistics analysis [38].

### Detection of Caspases Activation

A luminescence-based analysis was performed to investigate the activity of caspase 3/7 and caspase 9 using the Caspase-Glo 9 Assay and Caspase-Glo 3/7 Assay commercial kits (Promega Corporation, Fitchburg, WI, USA) as described previously [39]. Briefly, HT-29 cells (2 × 10^5^ cells/ml) were seeded overnight in a white-walled 96-well plate and treated with an IC_50_ dose of annomuricin E for 3, 6, 12, 24 and 48 h. After incubation, 100 μl of the caspase-Glo reagent was added to each well according to the manufacturer’s protocol. Luminescence, which represents the caspase activities, was measured using a luminescence microplate reader (Tecan Infinite 200 Pro).

### Multiple Cytotoxicity Assay

The simultaneous analysis of critical apoptosis markers, namely cell membrane permeability, cytochrome *c* leakage from the mitochondria, mitochondrial membrane potential (MMP) and total nuclear intensity, in HT-29 cells was performed using the Cellomics Multiparameter Cytotoxicity 3 Kit (Cellomics, Pittsburgh, PA, USA) as previously described in detail [40]. In brief, HT-29 cells (1 × 10^5^ cells/ml) were plated overnight in a 96-well plate and were exposed to an IC_50_ dose of annomuricin E for 12, 24 and 48 h. After incubation, the treated cells were stained with a cell permeability dye (FITC), a cytochrome *c* dye (Cy3), a mitochondrial membrane potential dye (Cy5) and a nuclear dye (Hoechst 33342), according to the vendor’s protocol. The plates were analyzed using a Cell Reporter cytofluorimeter system (Gentix/Molecular Devices, United Kingdom).

### Gene Expression Analysis of Bcl-2/Bax

The mRNA expression of two proteins, Bcl-2 and Bax, was quantified using real-time Q-PCR analysis as described previously with some modifications [41]. In brief, HT-29 cells at the exponential phase of growth were treated with annomuricin E at the IC_50_ concentration for 12, 24 and 48 h. The total RNA of treated cells was isolated using the RNeasy Plus Mini kit (Qiagen, Hilden, Germany) followed by the synthesis of the complementary DNA using the iScript cDNA synthesis kit (Biorad, Hercules, CA, USA). Q-PCR was performed on the StepOne PLUS real-time PCR machine (Applied Biosystems, Carlsbad, CA, USA). The β-actin housekeeping gene was used as a positive reference and was applied to normalize the target mRNA. The Q-PCR master mix was provided by Solaris Q-PCR Expression Assays (Thermo Fisher Scientific, Waltham, MA, USA) for the gene expression analysis of Bcl-2, AX-003307-00-0100; Bax, AX-003308-00-0100; and β-actin, AX-003451-00-0100.

### Immunofluorescence Analysis of Bcl-2/Bax

The perturbation in the protein expressions of Bcl-2 and Bax was investigated using immunofluorescence analysis as previously described in detail [37]. In brief, the HT-29 cells (5 × 10^4^ cells/ml) were seeded in a 96-well plate and exposed to the IC_50_ dose of annomuricin E for 12, 24 and 48 h. After washing the cells twice with PBS, they were fixed in 4% paraformaldehyde at 25°C for 20 min prior to blocking with blocking buffer (0.03% Triton X-100/PBS and normal serum) for 1 h. The cells were then supplemented with a primary antibody solution and incubated overnight at 4°C. After incubation, the cells were treated with Bcl-2 and Bax flurochrome-conjugated secondary antibody (Santa Cruz Biotechnology, Santa Cruz, CA, USA) for 1 h. The cells were then washed twice with PBS prior to staining with DAPI. The stained cells were examined using the Cell Reporter cytofluorimeter system.

### Statistical Analysis

Data from the rat study were reported as the means ± standard error of n animals per group. The experimental data were analyzed with one-way analysis of variance, followed by Tukey’s post hoc test using the SAS 9.1 statistical program (SAS Institute Inc., Cary, NC, USA). *In vitro* results were presented as the means ± standard error of the mean from three independent experiments. Statistical analysis was performed using the statistical package GraphPad Prism Version 5 (GraphPad Software Inc., San Diego, USA). One-way analysis of variance (Dunnett’s multiple comparison test) was used to distinguish the difference among groups. All values at *P*<0.05 were considered significant.

## Results and Discussion

### ACF Frequency

**Fig 2:**
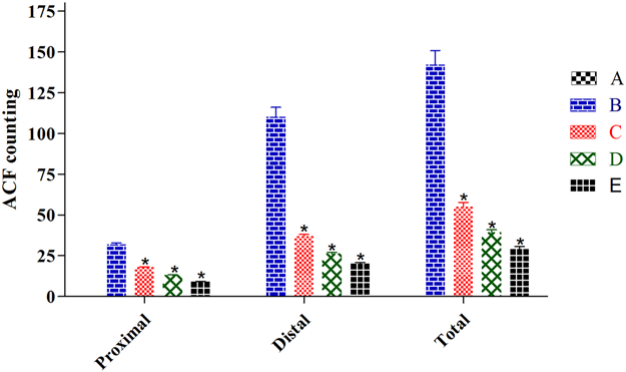
The number of ACF formed in proximal and distal parts of the colon. Tissue specimens were collected from five groups of rats: (A) negative control, (B) cancer control, (C) low dose of EEAML, (D) high dose of EEAML and (E) treatment control. Data are expressed as the means ± SEM of (n = 6/group). *P<0.05 compared with cancer control.

**Fig 3:**
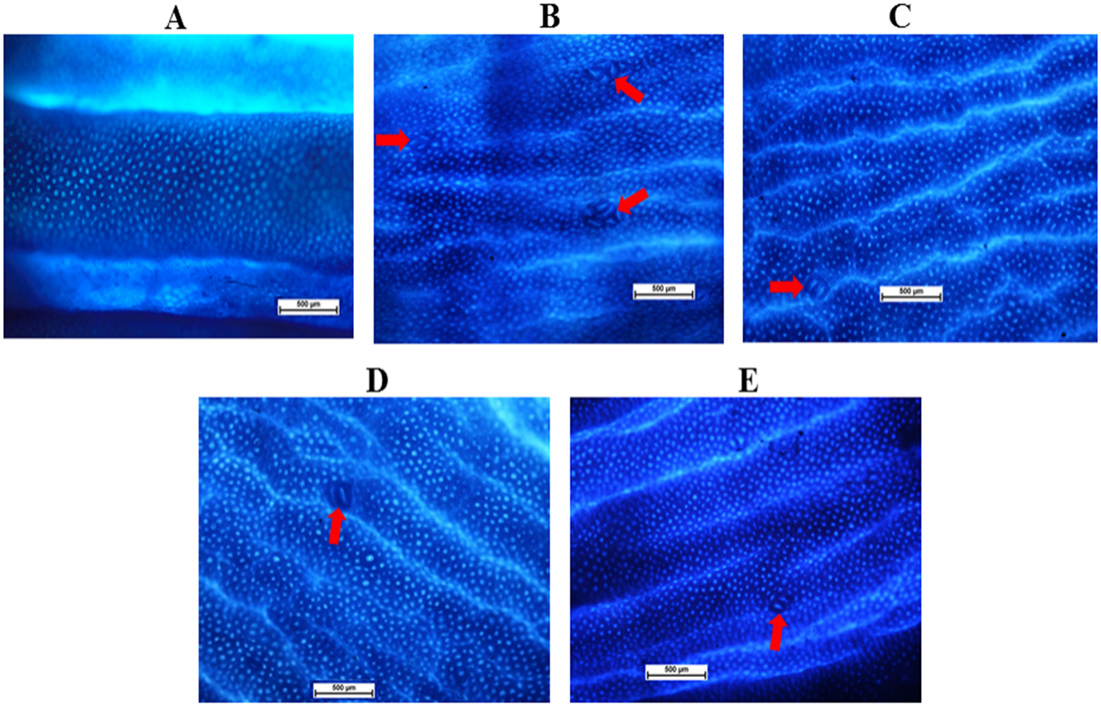
Topographical views of the colon mucosa. Tissue specimens were collected from five groups of rats: (A) negative control, (B) cancer control, (C) low dose of EEAML, (D) high dose of EEAML and (E) treatment control and were stained with methylene blue dye. The red arrows depict ACF in the colon mucosa. Scale bar: 500 μm.

To evaluate the effect of EEAML on suppressing colon carcinogenesis, ACF were employed as a biomarker to assess early stage AOM-induced colon cancer in rats. The incidence of ACF on the proximal and distal parts of the colon mucosa were analyzed with methylene blue staining immediately after the sacrifice of animals, and these data are shown in Fig 2 and Table 3. ACF were characterized by crypts with elevated sizes, altered luminal epithelia and easily discernible pericryptal zones. Topographical views of the stained colon specimens did not elicit any microscopic changes in the negative control group (Fig 3). Meanwhile, all rats injected with AOM developed ACF containing different numbers of crypts (Table 3). In agreement with previously published findings, ACF formation in the distal colon was significantly higher than the proximal colon [20,42]. Compared with the cancer control group, the administration of 5-FU or EEAML at 250 mg/kg or 500 mg/kg significantly suppressed the formation of ACF (79.5%, 61.2% and 72.5%, respectively). The doses used in this experiment were chosen based on the previous studies on the effect of different plant extracts against AOM-induced ACF formation [21,32]. A recent investigation on anticancer activity of *A*. *muricata* at a single dose of 300 mg/kg confirmed that these two doses would be appropriate for this study [43]. The respective investigation reported a similar reduction in ACF formation that demonstrated a potent anticancer activity of *A*. *muricata* leaves against 1, 2-dimethyl hydrazine-induced colon cancer, which was associated with an elevated apoptosis index [43]. In addition, another study on the breast tissues of female albino mice demonstrated that *A*. *muricata* leaves exhibited preventative effects against 7, 12-dimethylbenzene anthracene-induced breast cancer cell proliferation [44]. This growing body of experimental evidence supports the preventive effects of *A*. *muricata* leaves against cancer development and strongly supports the ethnomedicinal application of this plant.

**Table 3:** Distribution of aberrant crypt categories (1, 2, 3, 4 and more) in the colons of five groups of rats: (A) negative control, (B) cancer control, (C) low dose of EEAML, (D) high dose of EEAML and (E) treatment control.

**Group**	**No. of crypts per ACF**					
	**1 crypt**	**2 crypt**	**3 crypt**	**4 crypt and more**	**Total**	**Inhibition (%)**
**A**	0	0	0	0	0	-
**B**	33 ± 2.46	29 ± 1.89	48 ± 2.49	32 ± 2.32	142 ± 7.88	**-**
**C**	18 ± 0.92*	17 ± 0.68*	11 ± 0.66*	9 ± 0.48*	55 ± 2.32*	61.2
**D**	10 ± 0.65*	14 ± 0.59*	9 ± 0.52*	6 ± 0.25*	39 ± 1.48*	72.5
**E**	12 ± 0.26*	6 ± 0.45*	7 ± 0.78*	4 ± 0.18*	29 ± 1.40*	79.5

Data expressed as the means ± SEM of (n = 6/group).

*P<0.05 compared with cancer control.

### EEAML Induced the Down-Regulation of PCNA

Proliferating cell nuclear antigen (PCNA), originally known as DNA polymerase delta auxiliary protein, is a marker of cell proliferation because it is an indicator of a cell’s replication capability [45]. In mammalian cells, PCNA is involved in several metabolic pathways, including cell cycle, chromatin remodeling, DNA methylation, DNA repair, DNA synthesis and Okazaki fragment processing [46]. A number of clinical studies evaluating the inverse correlation between PCNA expression and cancer progression have led to the suggestion that the ratio of PCNA-positive cells provides a prognostic index for cancer [47,48].

**Fig 4:**
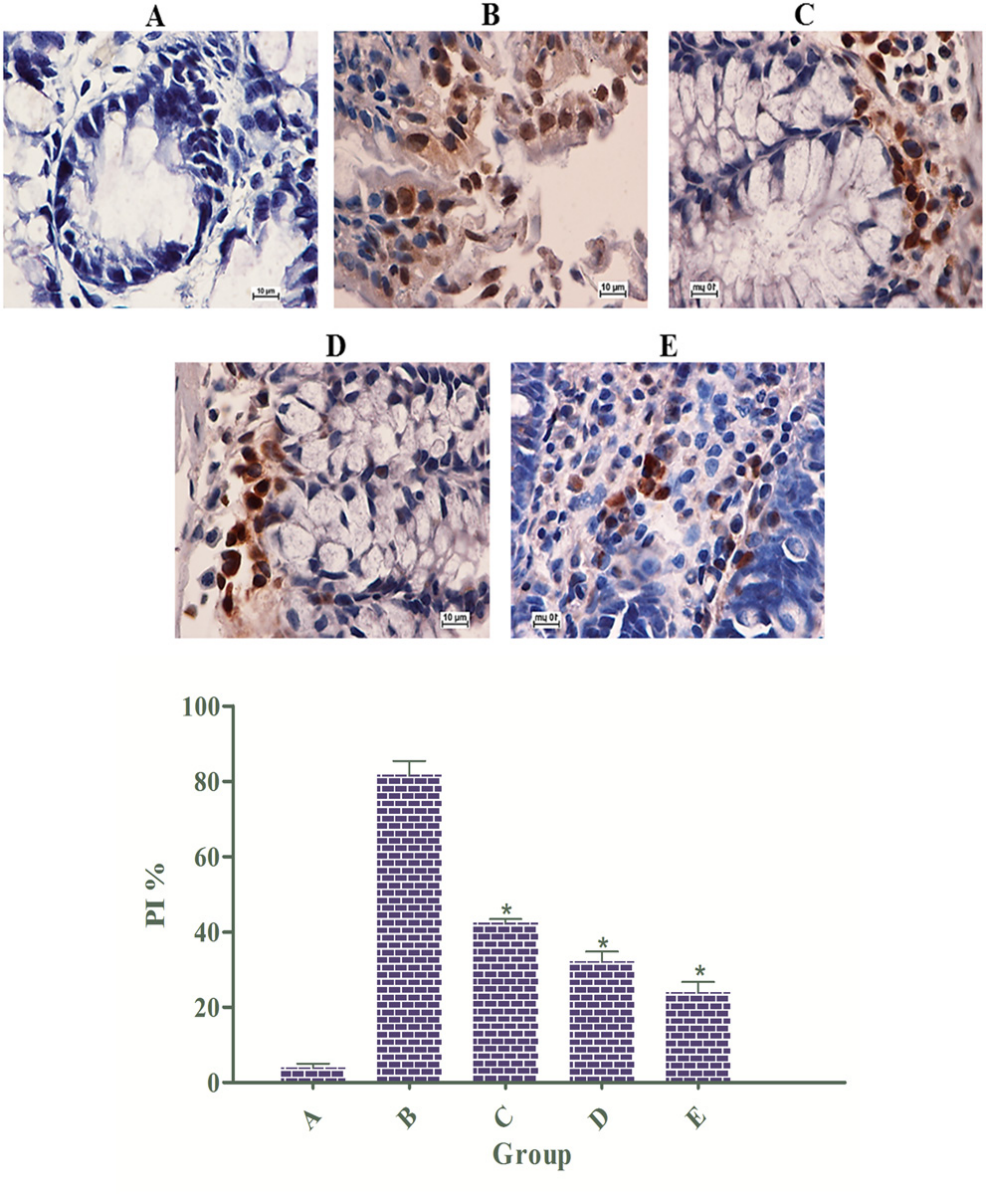
Immunohistochemical analysis of colon tissue sections for PCNA. Tissue specimens were collected from five groups of rats: (A) negative control, (B) cancer control, (C) low dose of EEAML, (D) high dose of EEAML and (E) treatment control. Quantitative analysis of immunopositivity shown as brown staining demonstrated a significant down-regulation of PCNA in groups C-E compared with the cancer control group. Data are expressed as the means ± SEM of (n = 6/group). *P<0.05 compared with the cancer control group. Scale bar: 10 μm.

Because an elevated rate of proliferation is a critical hallmark for ACF formation in colon tissues, we investigated PCNA expression using immunostaining. Microscopic examinations of colon tissue sections clearly revealed an elevated level of PCNA-positive cells in the cancer control group (PI: 81%) compared with the negative control group (PI: 4%). The rats treated with 5-FU (PI: 24%) exhibited a significantly lower number of positive cells compared with the rats treated with AOM, and similar results were observed in rats treated with EEAML at doses of 250 mg/kg (PI: 42%) and 500 mg/kg (PI: 31%) (Fig 4).

Oncology studies have proven that aberrant proliferation of epithelial cells is one of the early indicators of pre-neoplasia [49,50]. Deschner et al. [51] earlier reported that the administration of chemical carcinogens to animals leads to an extensive proliferation zone with an elevated labeling index. The results of the present study revealed the potential role of PCNA down-regulation on the protective effects of EEAML against induced colon cancer. We found that EEAML administration caused an attenuated proliferation zone and a lower labeling index. A similar decline in PCNA expression was reported earlier in an investigation that showed effective chemoprevention against AOM-induced colon cancer in rats [52]. Based on our results, EEAML is postulated to suppress ACF formation through perturbations in cell proliferation pathways.

### EEAML Induced the Up-Regulation of Bax and the Down-Regulation of Bcl-2

The Bcl-2 family of proteins, including pro-apoptotic and anti-apoptotic proteins, contains a total of 25 genes and plays a pivotal role in the control and regulation of mitochondria-mediated apoptosis [53,54]. The pro-apoptotic protein Bax mediates the leakage of pro-apoptotic factors, including cytochrome *c*, Ca^2+^ and Smac/DIABLO, into the cytosol through dimerization and translocation to the outer mitochondrial membrane [13]. Anti-apoptotic proteins, including Bcl-2, in turn suppress the function of apoptosis mediators [55].

**Fig 5:**
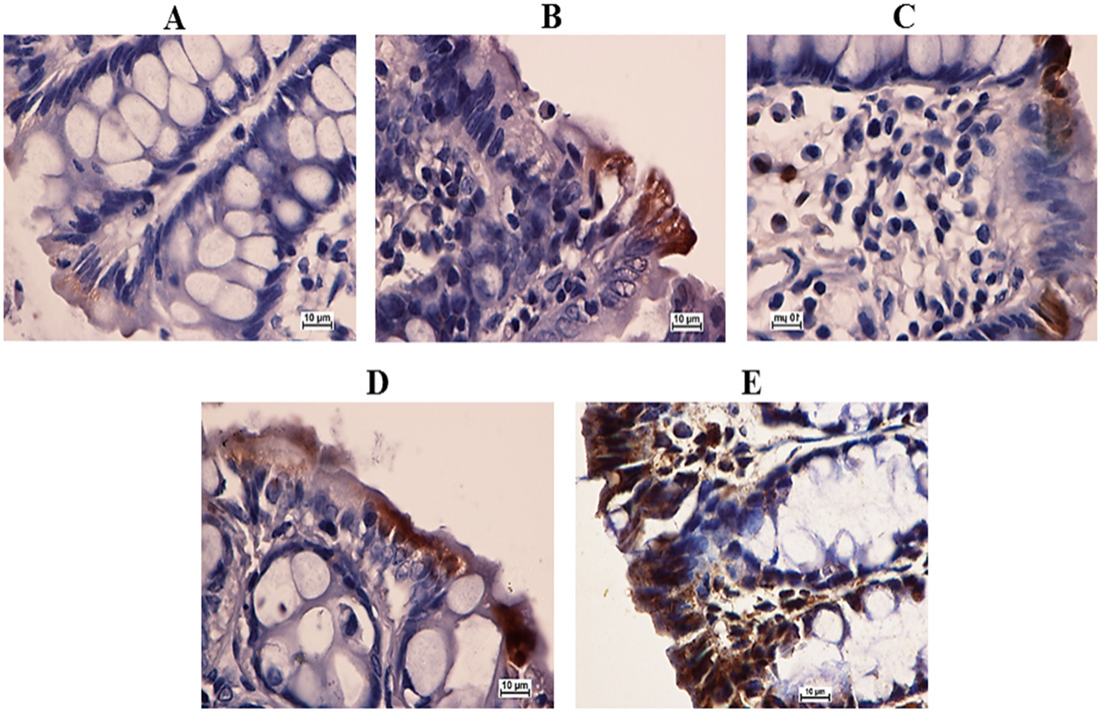
Expression of Bax in colon tissue sections. Tissue specimens were collected from five groups of rats (n = 6/group) and were analyzed using immunohistochemistry: (A) negative control, (B) cancer control, (C) low dose of EEAML, (D) high dose of EEAML and (E) treatment control. The up-regulation of Bax in groups C-E is shown as brown staining. Scale bar: 10 μm.

**Fig 6:**
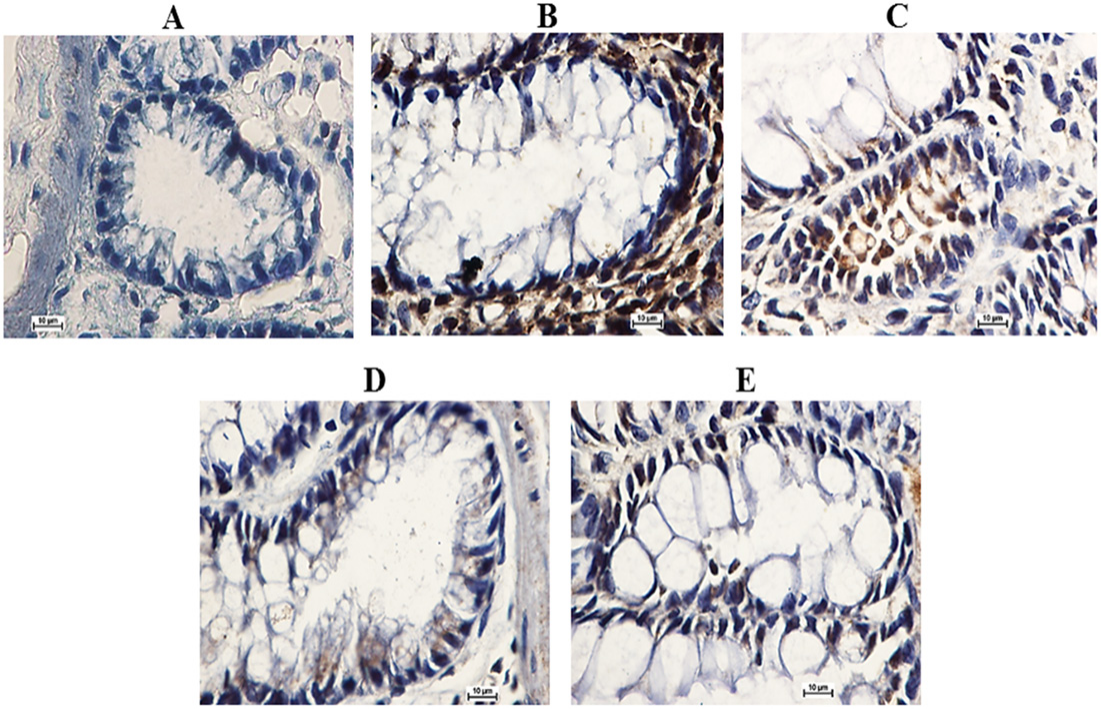
Immunohistochemical analysis of colon tissue sections for Bcl-2. Tissue specimens were collected from five groups of rats (n = 6/group): (A) negative control, (B) cancer control, (C) low dose of EEAML, (D) high dose of EEAML and (E) treatment control. Immunopositivity shown as brown staining revealed the down-regulation of Bcl-2 in groups C-E. Scale bar: 10 μm.

In the present study, immunohistochemical evaluation demonstrated the up-regulation of Bax and down-regulation of Bcl-2 in colon tissues after treatment with EEAML (both doses) and 5-FU. Our results showed that an accumulation of Bax in the colon tissues of rats treated with EEAML was comparable to that of rats treated with 5-FU (Fig 5). As shown in Fig 6, Bcl-2 expression in the cancer control group was noticeably higher than the negative control group, representing the suppression of apoptosis among colon cells. In rats treated with EEAML at doses of 250 mg/kg and 500 mg/kg and 5-FU, an accumulation of Bcl-2 protein in colon tissues was markedly decreased. Administration of EEAML (500 mg/kg) and 5-FU decreased Bcl-2 protein expression to approximately the level of the negative control group.

Previous studies have reported that high Bax protein expression may augment the median survival among cancer patients [56]. In addition, a deficiency in Bax protein has a strong impact on tumor clonal evolution [57]. The results of our present study demonstrate that EEAML has the potential to induce apoptosis in colon cells that are susceptible to AOM damage. This *in vivo* observation agreed with our previous *in vitro* study by illustrating the up-regulation of Bax and the down-regulation of Bcl-2 in HT-29 cells treated with EEAML [30].

### EEAML Augmented Enzymatic Antioxidants Activities

As an aggressive factor, reactive oxygen species (ROS) play a pivotal role in the pathogenesis of colorectal cancer [58]. The production of reactive oxygen species (ROS) are part of the normal metabolism in the human body, and cellular antioxidants containing enzymatic and non-enzymatic scavengers maintain ROS at their physiological levels [59]. Nonetheless, an extensive generation of ROS, including hydrogen radicals, hydrogen peroxide and superoxide anions, causes oxidative stress, which leads to metabolic impairments and irreversible cell damages [60]. SOD, the first scavenging barrier against ROS, converts the superoxide to hydrogen peroxide, which is subsequently degraded to water and oxygen by CAT [61]. The degradation of lipid peroxides to hydroxyl lipids and water is mediated by GPx through oxidation of glutathione to glutathione disulfide [62,63].

**Fig 7:**
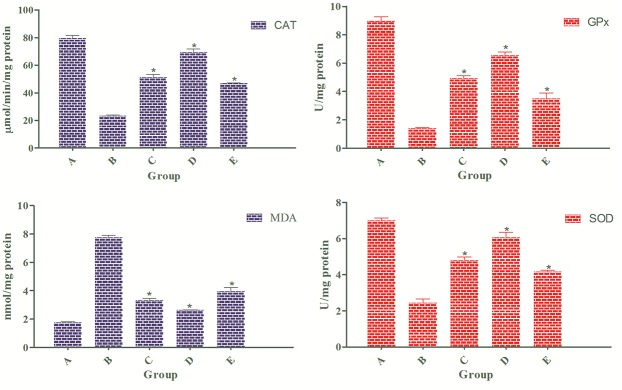
Level of CAT, GPx, MDA and SOD in colon tissue homogenates. Samples were collected from five groups of rats: (A) negative control, (B) cancer control, (C) low dose of EEAML, (D) high dose of EEAML and (E) treatment control. Data are expressed as the means ± SEM of (n = 6/group). *P<0.05 compared with cancer control.

The activities of antioxidant enzymes were significantly reduced in the AOM-treated group compared with the negative control group (Fig 7). However, EEAML supplementation at both doses significantly restored the levels of these enzymes towards normal values. As expected, EEAML showed a greater antioxidant defense than 5-FU. A number of earlier *in vitro* and *in vivo* studies have demonstrated that the leaves of *A*. *muricata* possess significant antioxidant potential [64–66]. Moreover, the leaves elicited noticeable defensive activities against acute and chronic inflammation in rats through suppressive effects on the secretion of proinflammatory cytokines [67]. Immunological studies have led to the suggestion that concomitant administration of chemotherapeutic agents and antioxidant drugs counteract chemotherapy toxicity and enhance the survival rate among cancer patients [68,69]. Therefore, an establishment of anticancer agents with innate antioxidant defense may result in the discovery of new generations of anticancer drugs.

### EEAML Suppressed Lipid Peroxidation

Excessive ROS generation results in the production of lipid radicals and rearrangements of unsaturated lipids, leading to the formation of different degraded metabolites, including alkenes, lipid hydroperoxides and MDA, which eventually disrupt the integrity of membrane lipids [70,71]. MDA, a major metabolite of this process, is an easy indicator of lipid peroxidation and oxidative stress [72]. As a carcinogenic agent, AOM causes lipid peroxidation as a result of oxidative stress [73], which was observed in our study after administration of AOM to the cancer control group (Fig 7). This result appears to be in line with previous reports that plasma and tissue MDA concentrations are markedly elevated in patients suffering from colorectal cancer [74,75]. As expected, because of the augmentation in the enzymatic and antioxidant activities, EEAML treatment at both doses significantly reduced MDA formation in colon tissues, and this reduction was stronger than the reduction found after treatment with 5-FU. This result confirmed the protective effects of EEAML against oxidative stress in colon tissues, which was reflected by reduced MDA production.

### Isolation of the Bioactive Compound, Annomuricin E

**Fig 8:**
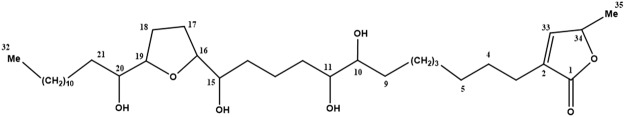
Chemical structure of annomuricin E.

The dried leaves of *A*. *muricata* were extracted with ethyl acetate at room temperature. After concentrating the solution until dry, the ethyl acetate extract was obtained. The ethyl acetate extract was fractionated by chromatography on a silica gel 60 column, which yielded six fractions. Fraction 3 (3.9 g) was further purified on a micro column followed by preparative TLC and finally preparative HPLC using an ODS C-18 column and a PDA detector to obtain annomuricin E (Fig 8), which was identified by 1D and 2D NMR, mass spectrometry and other physical properties that were then compared with reported data [76].

The annonaceous acetogenins, a series of C-35/C-37 fatty acid derivatives, are a class of natural products that are uniquely isolated from the Annonaceae family [77]. The isolation of more than 500 annonaceous acetogenins from different parts of plants in this family has been performed for more than 27 years [78]. This hyperbioactive group of natural products exhibits a variety of bioactivities, including anticancer, antiparasitic, immunosuppressive and insecticidal effects [79,80]. Due to the broad spectrum of bioactivities in annonaceous acetogenins, modified analogues and mimics of these compounds were synthesized to substantiate the ideas regarding the mechanisms of these compound [78]. Previous studies have reported that they are potent suppressors of complex I (NADH, ubiquinone oxidoreductase) in insect and mammalian mitochondrial electron transport systems and of NADH oxidase in the plasma membrane of cancer cells [81–83]. Hence, further studies on annonaceous acetogenins may lead to the establishment of new generations of anticancer drugs.

### Annomuricin E Suppressed the Proliferation of HT-29 Cells

Annomuricin E was investigated for its suppressive effect against HT-29 colon cancer cells and CCD841 normal colon cells using the MTT assay. As shown in Table 4, the IC_50_ value of annomuricin E on HT-29 cells was 5.72 ± 0.41 μg/ml, 3.49 ± 0.22 μg/ml and 1.62 ± 0.24 μg/ml after 12, 24 and 48 h treatments, respectively, which were comparable with the suppressive potential of 5-FU as a standard anticancer drug. When compared with HT-29 cells, annomuricin E was far less cytotoxic to the normal cells, as revealed by the relatively high IC_50_ value on CCD841 (32.51 ± 1.18 μg/ml for 48 h). These results are in line with a previous report that showed the cytotoxic effect of annomuricin E against six different human cancer cells with selectivities toward PACA-2 (a pancreatic carcinoma cell line) and HT-29 cells [76]. In addition, earlier studies have shown that acetogenins are potentially effective against multidrug resistant cancer cell lines [84,85].

**Table 4:** Cytotoxic effects of annomuricin E and 5-FU on the proliferation of CCD841 and HT-29 cells after 12, 24 and 48 h of treatment.

Cell line	IC_50_ (μg/ml)					
	Annomuricin E 12 h	5-FU 12 h	Annomuricin E 24 h	5-FU 24 h	Annomuricin E 48 h	5-FU 48 h
HT-29	5.72 ± 0.41	4.85 ± 0.38	3.49 ± 0.22	2.96 ± 0.43	1.62 ± 0.24	1.50 ± 0.17
CCD841	64.32 ± 3.76	58.50 ± 2.09	47.10 ± 0.47	44.35 ± 2.25	32.51 ± 1.18	36.32 ± 0.43

The IC_50_ values represent the means ± SEM of three independent experiments.

### Annomuricin E Induced LDH Leakage in HT-29 Cells

**Fig 9:**
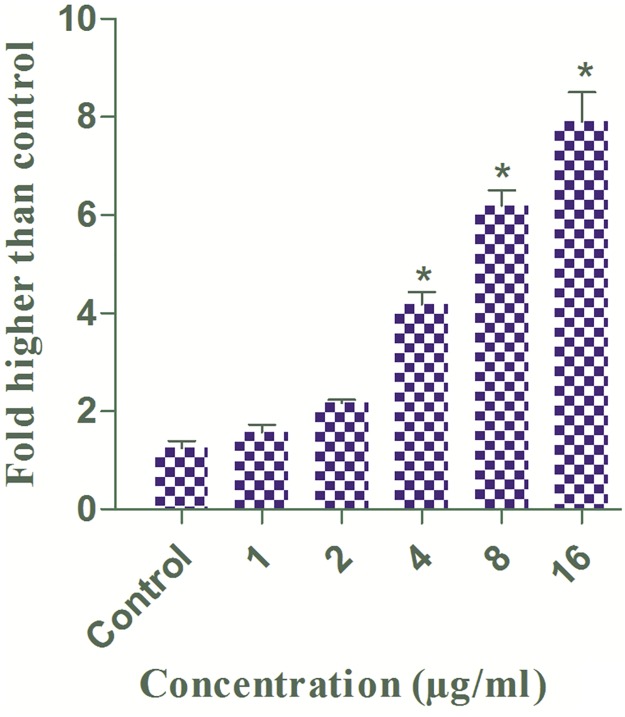
Effects of annomuricin E on LDH leakage formation in HT-29 cells. Cells were exposed to 0.1% vehicle DMSO (control) and annomuricin E at different concentrations for 24 h. The treated HT-29 cells showed a significant LDH release at 4 to 16 μg/ml concentrations compared with the control. The data represent the means ± SEM of three independent experiments. *P<0.05 compared with the control.

Because any irreversible membrane damage to cells causes a leakage of LDH from the cytosol, the level of this stable cytosolic enzyme in cellular culture supernatants is a simple and quick assay to determine the cellular cytotoxicity [86]. The cytotoxic effect of annomuricin E against HT-29 cells was further substantiated by an LDH assay. As depicted in Fig 9, the control cells treated with 0.1% vehicle DMSO showed a low level of LDH release after 24 h of treatment. In contrast, LDH leakage modestly increased with the presence of annomuricin E at concentrations of 1 and 2 μg/ml. Meanwhile, treatment of concentrations from 4 to 16 μg/ml led to a significant LDH release compared with the control. The significant LDH leakage from HT-29 cells was shown at concentrations as low as 4 μg/ml, which was compatible with the 24-h IC_50_ value of annomuricin E (3.49 ± 0.22 μg/ml) against HT-29 cells.

### Cell Cycle Arrest at G_1_ Induced by Annomuricin E

**Fig 10:**
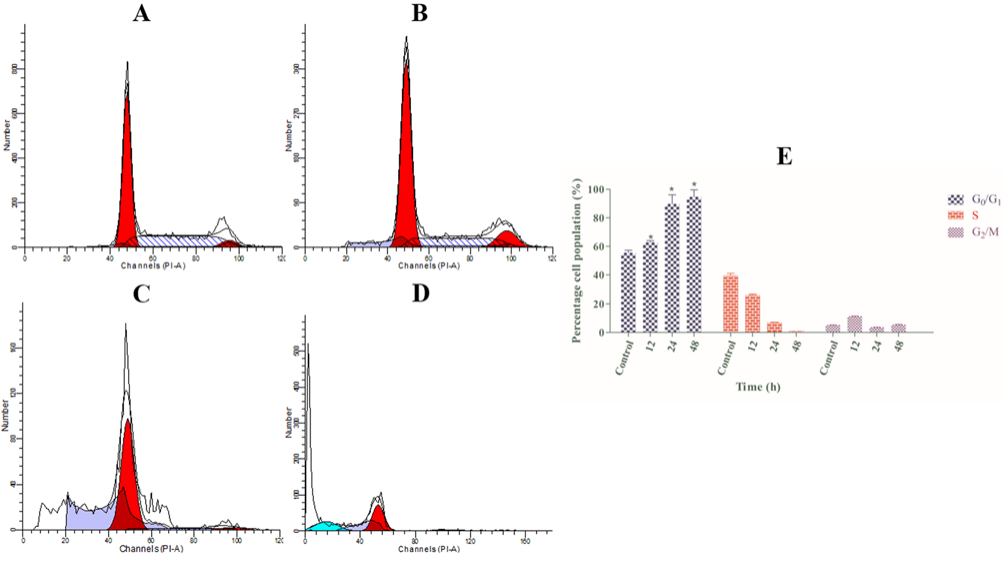
Effect of annomuricin E on cell cycle distribution in HT-29 cells. Cells were treated with (A) 0.1% vehicle DMSO (control) for 48 h and annomuricin E at the IC_50_ concentration for (B) 12, (C) 24 and (D) 48 h. After staining the cells with PI, the DNA contents were monitored using flow cytometry. (E) The representative bar chart shows the significant induction of G_1_ cell cycle arrest by annomuricin E after 12 h of treatment. The data represent the means ± SEM of three independent experiments. *P<0.05 compared with the control.

Cancer progression is often associated with irregularities in cell cycle function [87]. A growing body of experimental evidence supporting the concomitant involvement of cell cycle suppression and apoptosis has stimulated widespread attention to phytochemicals with cell-cycle modulatory effects [15,88]. Hence, we first evaluated whether the suppressive effect of annomuricin E was accompanied by a block in the cell cycle using PI staining and flow cytometry analysis. As illustrated in Fig 10, the augmented accumulation of HT-29 cells in the G_1_ phase was initiated after 12 h of treatment with annomuricin E, and this accumulation of cells in the G_1_ phase continued in a time-dependent manner. After 24 and 48 h, the percentage of HT-29 cells treated with annomuricin E that were arrested at the G_1_ phase reached 89.65% and 94.60%, respectively. This was accompanied by a concurrent decline in the S and G_2_/M cell populations compared with the control. These results indicated that annomuricin E arrested HT-29 cells at the G_1_ phase. An earlier study on annonacin, an annonaceous acetogenin from the seeds of *Annona reticulata*, also showed the induction of cell cycle arrest in T24 bladder cancer cells at the G_1_ phase through the activation of p21 [89].

### Phosphatidylserine Externalization Induced by Annomuricin E

**Fig 11:**
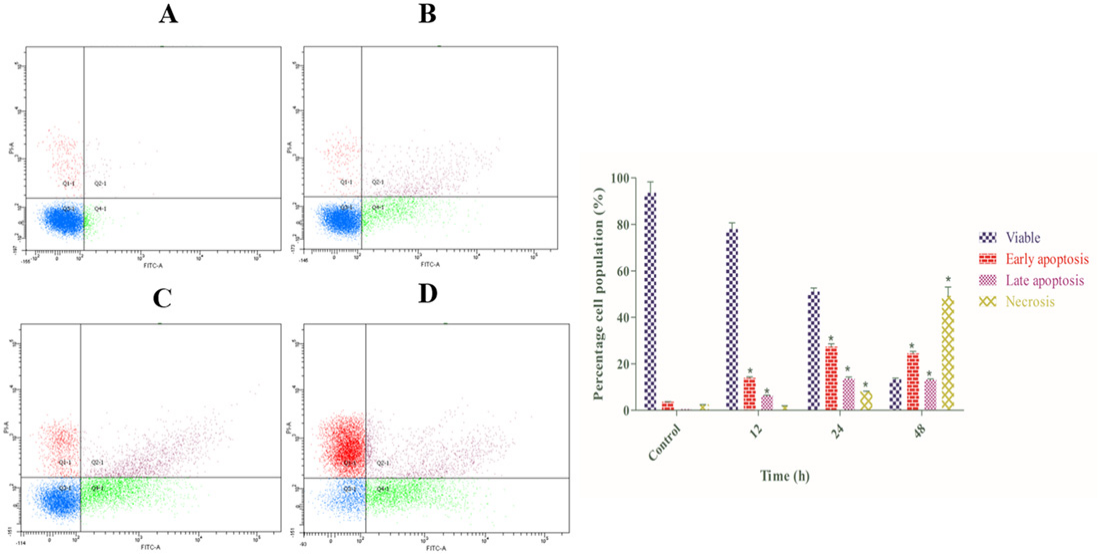
Effect of annomuricin E on apoptosis in HT-29 cells via quadrant statistics. After treatment with annomuricin E (IC_50_ concentration) for (B) 12, (C) 24 and (D) 48 h, the cells were double stained with Annexin V-FITC/PI and monitored using flow cytometry. Cells treated with 0.1% vehicle DMSO were employed as the (A) control treatment. (E) The representative bar chart depicted the percentages of early apoptotic, late apoptotic and necrotic cells. The data represent the means ± SEM of three independent experiments. *P<0.05 compared with the control.

As one of the biochemical characterizations of apoptosis, a transverse redistribution of phosphatidylserine (PS) on the outer plasma membrane arises during early apoptosis [90]. A fluorescent probe of Annexin V-FITC is a recombinant protein with a high affinity for externalized PS [91]. To gain insight into the mechanism through which annomuricin E induces its cytotoxic effects, HT-29 cells were stained with Annexin V-FITC/PI and analyzed using flow cytometry. In cells treated with 0.1% vehicle DMSO (control), only 3.6% and 0.5% of cells were in early (Annexin V^+^/PI^−^) and late (Annexin V^+^/PI^+^) apoptosis after 48 h, respectively (Fig 11). However, the percentage of early and late apoptotic cells were significantly increased to 13.9% and 6.9%, respectively, after being treated with annomuricin E (IC_50_ concentration) for 12 h. The percentages of early and late apoptotic populations peaked at 24 h with values of 27.3% and 13.6%, respectively, and were reduced slightly at 48 h. This was reduction was associated with a significant elevation in the number of necrotic cells (Annexin V^−^/PI^+^) at 24 and 48 h. This increase in necrotic cells can be explained by the long exposure of annomuricin E to HT-29 cells that allowed the cells to enter secondary necrosis from primary apoptosis, increasing the number of dead cells. These data showed that annomuricin E caused its cytotoxic effects through the induction of apoptosis in HT-29 cells.

### Caspase Activation Induced by Annomuricin E

**Fig 12:**
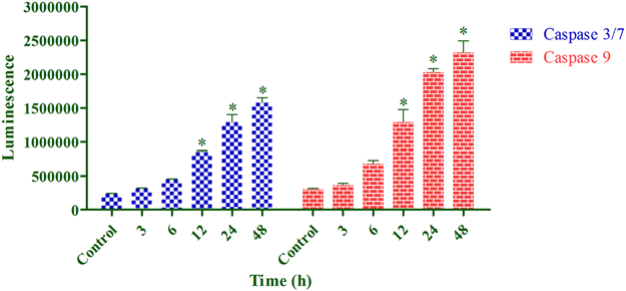
Effect of annomuricin E on caspase 3/7 and caspase 9 activities in HT-29 cells using bioluminescent analysis. Cells were treated with the IC_50_ concentration of annomuricin E for 3, 6, 12, 24 and 48 h. The activities of both caspase were significantly elevated after 12 h of treatment. Cells treated with 0.1% vehicle DMSO were employed as the control treatment. The data represent the means ± SEM of three independent experiments. *P<0.05 compared with the control.

The energy-dependent process of apoptosis relies heavily on the family of caspases, or cysteinyl aspartate proteinases, to hierarchically convert the initiating cellular stimuli to the final cell demise [14]. Caspases, which are initially synthesized as inactive proforms, consist of three main classes: inflammatory (caspase-1, -4, -5), initiator (caspase-2, -8, -9, -10) and executioner or effector caspases (caspase -3, -6, -7) [54]. A complex cascade of caspase-dependent events in the apoptosis process is triggered by initiators and is finalized by effectors that mediate the typical biochemical modifications during apoptosis execution [92]. To determine whether annomuricin E-induced apoptosis in HT-29 cells is mediated through caspases, the activities of caspase 9 and caspase 3/7 were investigated using bioluminescent analysis. In this assay, the luminescent intensity is proportional to the activation of caspases. As shown in Fig 12, both caspase 9 and caspase 3/7 were activated in a time-dependent manner after exposure to annomuricin E at the IC_50_ concentration. After 12 to 48 h of treatment, the activities of both caspases were significantly elevated, suggesting that annomuricin E-induced apoptosis occurs through the involvement of caspase 9 and caspase 3/7 activation.

### Mitochondria-Initiated Events Induced by Annomuricin E

**Fig 13:**
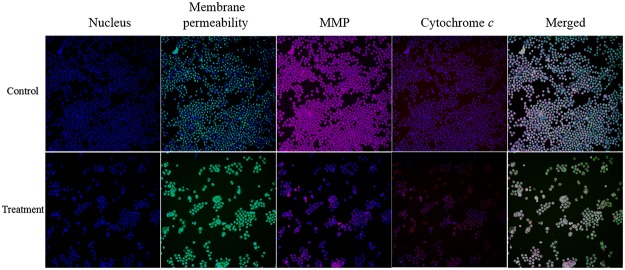
Images of HT-29 cells treated with annomuricin E at the IC_50_ concentration for 24 h. The treated cells were stained with different and specific dyes for the detection of total nuclear intensity, cell membrane permeability, MMP and cytochrome *c* release. Cells treated with 0.1% vehicle DMSO were employed as the control treatment.

**Fig 14:**
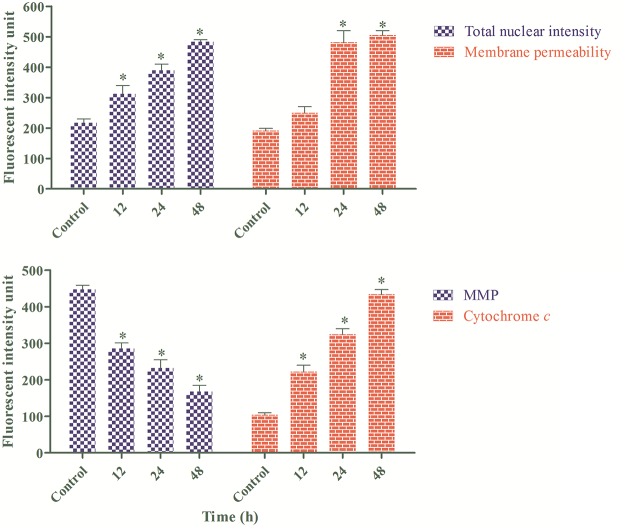
Representative bar charts of the multiple cytotoxicity assay. After 12 h of treatment with annomuricin E at the IC_50_ concentration, the total nuclear intensity, MMP and cytochrome *c* release were significantly elevated compared with the control. However, cell membrane permeability showed a significant increase only after 24 h. Cells treated with 0.1% vehicle DMSO were employed as the control treatment. The data represent the means ± SEM of three independent experiments. *P<0.05 compared with the control.

As a convergent center of internal apoptotic stimuli, mitochondria play a pivotal role in the intrinsic pathway of apoptosis. Depletion of MMP leads to the opening of mitochondria permeability transition pores and the further release of pro-apoptotic proteins, such as cytochrome *c*, from the mitochondria to the cytosol, resulting in the formation of the apoptosome and the activation of caspase 9 [93]. The aforementioned results revealed that caspase 9 activation occurred during the exposure of HT-29 cells to annomuricin E. Subsequently, we examined the mitochondria-initiated events in treated HT-29 cells using a cell reporter system. As shown in Fig 13, the number of cells was considerably reduced after exposure to annomuricin E at the IC_50_ concentration. Four florescent markers were used to monitor the changes in total nuclear intensity, cell membrane permeability, MMP and cytochrome *c* release of the treated cells in a time-course experiment. A 12-h treatment of annomuricin E induced a significant collapse in MMP associated with an increase in cytochrome *c* leakage from the mitochondria in a time-dependent manner (Fig 14). The total nuclear intensity represents pyknosis as a result chromatin condensation, which is the most characteristic property of apoptosis [54], and was significantly elevated at 12, 24 and 48 h (Fig 14). In addition, the cell membrane permeability of HT-29 cells was significantly increased only at the later stages of treatment (24 and 48 h). These results suggest that annomuricin E caused the dissipation of MMP and the leakage of cytochrome *c* from mitochondria, which resulted in the activation of caspase 9.

### Bax Up-Regulation and Bcl-2 Down-Regulation Induced by Annomuricin E

**Fig 15:**
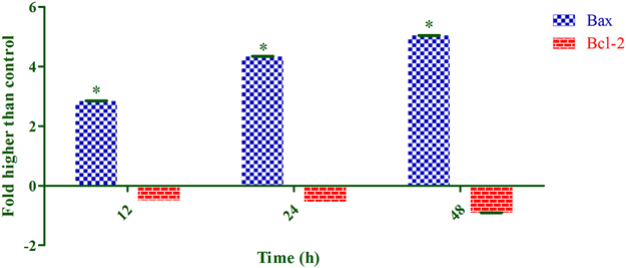
Effect of annomuricin E on Bax and Bcl-2 mRNA expression was assessed using Q-PCR analysis. The housekeeping gene β-actin was used for the normalization of the mRNA expression. The result depicted a time-dependent upregulation of Bax and down-regulation of Bcl-2 after treatment with annomuricin E at the IC_50_ concentration. The data represent the means ± SEM of three independent experiments. *P<0.05 compared with the control.

**Fig 16:**
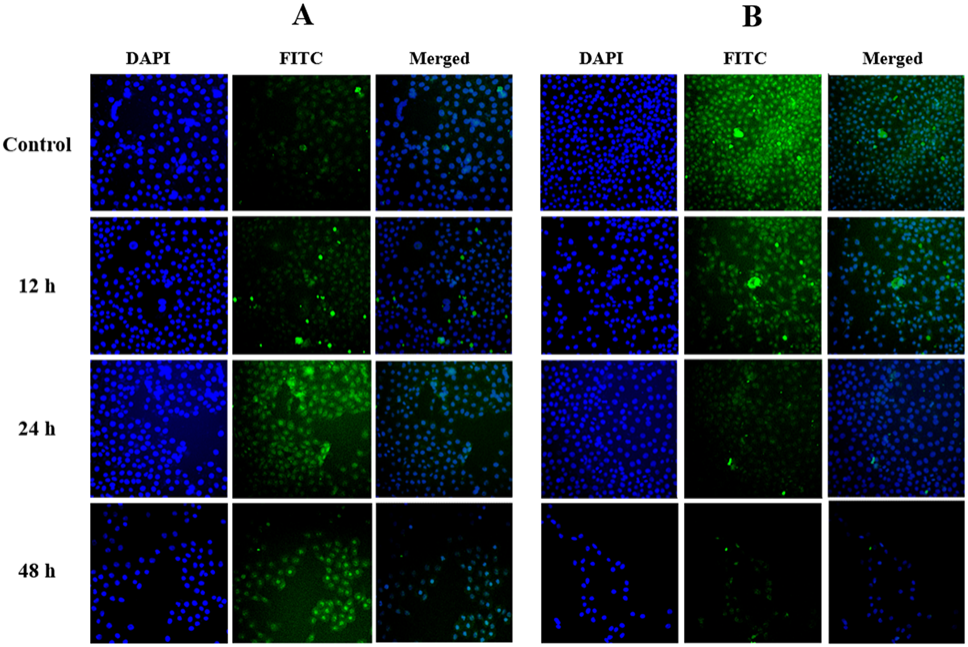
Immunofluorescence analysis of (A) Bax and (B) Bcl-2 protein expression in HT-29 cells. Cells were treated with annomuricin E at the IC_50_ concentration for 12, 24 and 48 h and were stained with DAPI and Bax/Bcl-2 antibodies conjugated to FITC. Cells treated with 0.1% vehicle DMSO were employed as the control treatment. As the number of cells reduced in a time-dependent manner, the fluorescent intensity showed a marked upregulation and down-regulation for Bax and Blc-2 proteins, respectively.

**Fig 17:**
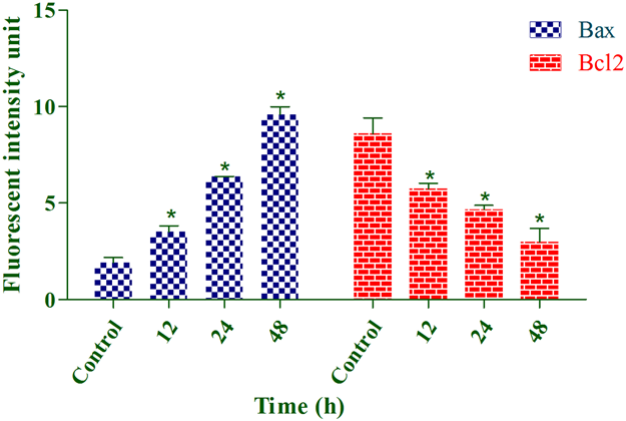
Representative bar charts of Bax and Bcl-2 immunofluorescence analysis. Annomuricin E at IC_50_ concentration induced significant upregulation of Bax and down-regulation of Bcl-2 after 12 h. Cells treated with 0.1% vehicle DMSO were employed as the control treatment. The data represent the means ± SEM of three independent experiments. *P<0.05 compared with the control.

Because annomuricin E elicited the ability to interfere with MMP in HT-29 cells, we raised the possibility of Bax and Bcl-2 involvement in annomuricin E-induced apoptosis. Hence, the expression of Bax and Bcl-2 was investigated at both the mRNA and protein levels using Q-PCR and immunofluorescence analysis, respectively. As shown in Fig 15, the mRNA expression of the Bax protein was significantly and time-dependently elevated after 12 h treatment and reached an approximately 5-fold higher level after 48 h. In spite of Bax up-regulation, the mRNA expression of the anti-apoptotic protein Bcl-2 was time-dependently reduced from 12 to 48 h. Immunofluorescence analysis demonstrated that the number of HT-29 cells treated with annomuricin E decreased in a time-dependent manner after 12, 24 and 48 h, as illustrated by the blue fluorescent staining of DAPI, which identifies all cell nuclei (Fig 16). The time-dependent reduction in the number of surviving cells was accompanied with a distinct increase in the fluorescent intensity of FITC dye (green) that represented Bax protein expression, which reached a value approximately 10-fold higher than the control after 48 h (Fig 17). Bcl-2 protein expression also significantly and dose-dependently reduced compared with the control. The perturbations in Bax and Bcl-2 expression at the mRNA and protein level substantiated the idea that annomuricin E-induced apoptosis was through the mitochondria-mediated pathway.

## Conclusions

Overall, we provided evidence that the ethyl acetate extract of *A*. *muricata* leaves has the potential to suppress the AOM-induced development of ACF in rats. The data substantiated the traditional use of *A*. *muricata* leaves against cancer and tumors. The suppressed formation of ACF in rats after EEAML oral administration was accompanied with down-regulation of PCNA and Bcl-2 proteins and up-regulation of Bax protein in the colon tissue, indicating a possible mechanism at the molecular level. The reported pharmacological effect of the *A*. *muricata* leaves may be partially due to the presence of annomuricin E. This acetogenin suppressed the proliferation of HT-29 cells selectively and induced apoptosis that was associated with G_1_ cell cycle arrest and mitochondria-mediated pathways.

## Supporting Information

S1 Fig ^1^H NMR (500 MHz) and ^13^C NMR (125 MHz) spectral data of annomuricin E in CDCl_3_ (δ in ppm, *J* in Hz). (DOCX)
