# Supplementary material for: The Chemopotential Effect of Annona muricata Leaves against Azoxymethane-Induced Colonic Aberrant Crypt Foci in Rats and the Apoptotic Effect of Acetogenin Annomuricin E in HT-29 Cells: A Bioassay-Guided Approach
Source: PLoS One. 2015 Apr 10;10(4):e0122288. doi: 10.1371/journal.pone.0122288 (PMC4393181; doi:10.1371/journal.pone.0122288)

^1^H NMR of annomuricin E


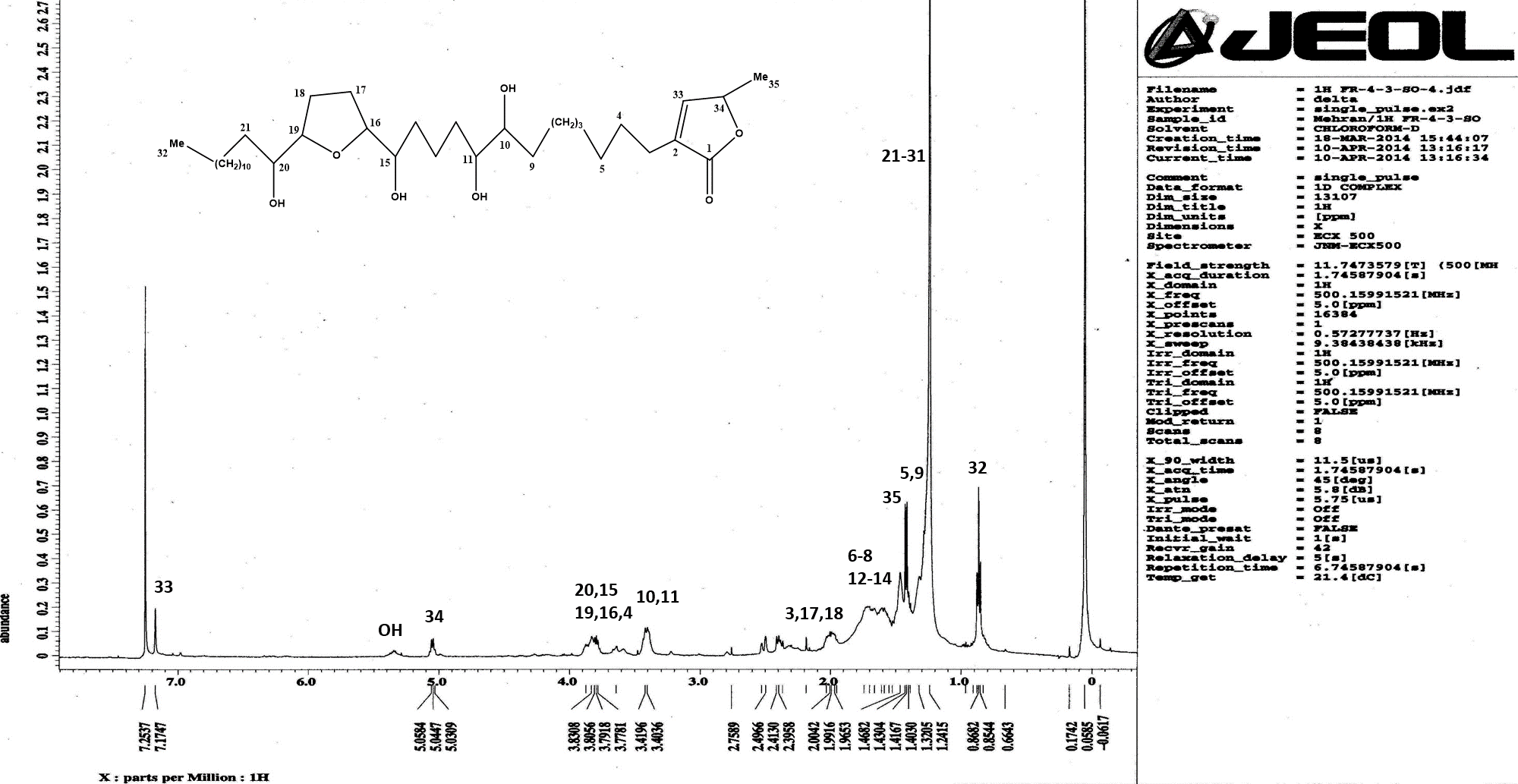


^13^C NMR of annomuricin E


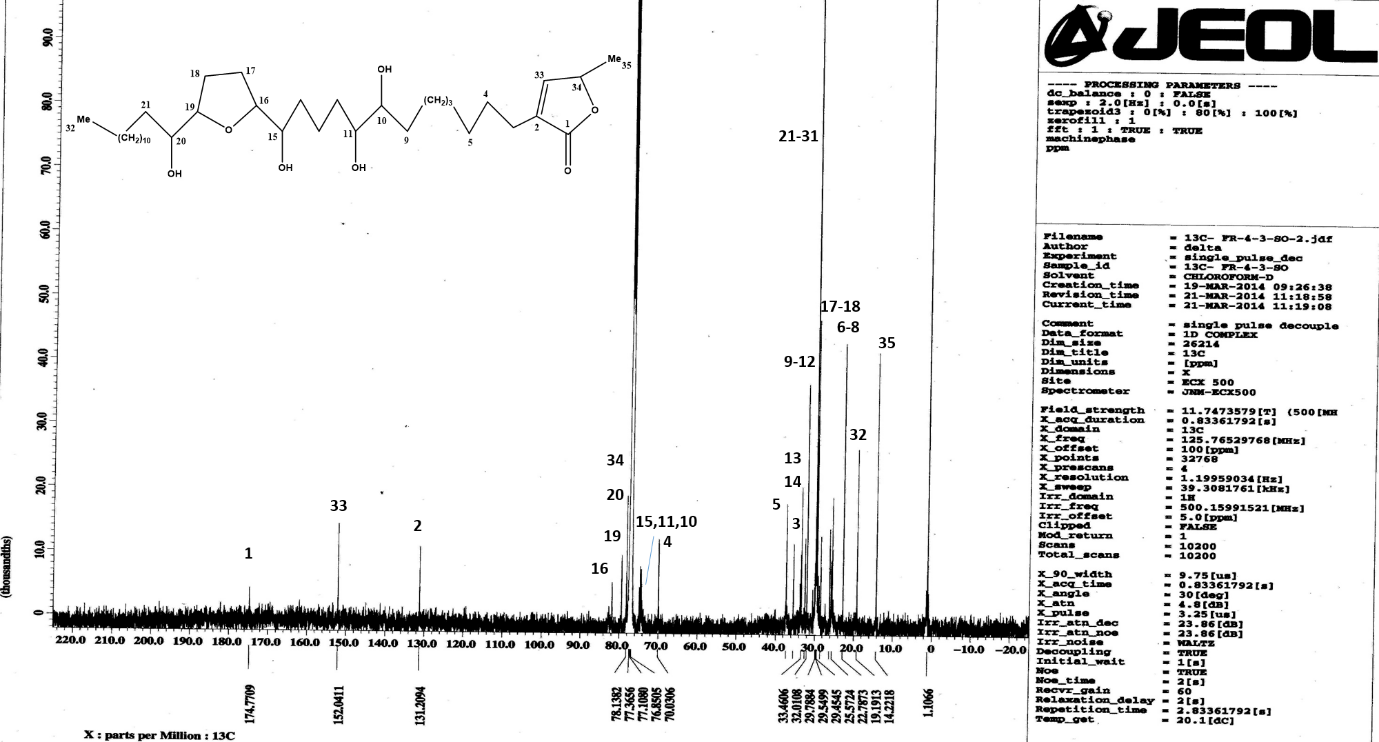


DEPT 135 of annomuricin E


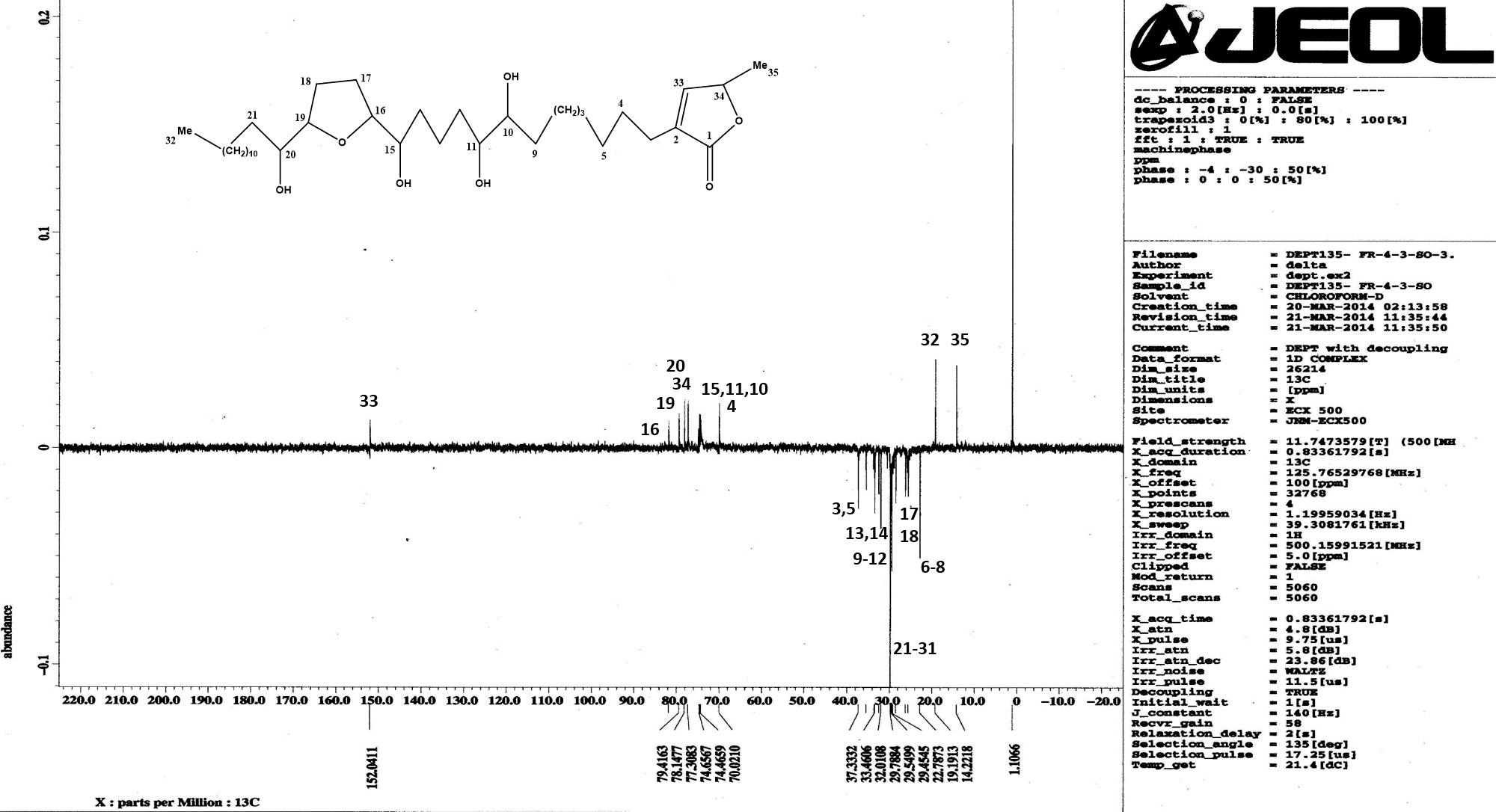


HMQC NMR of annomuricin E


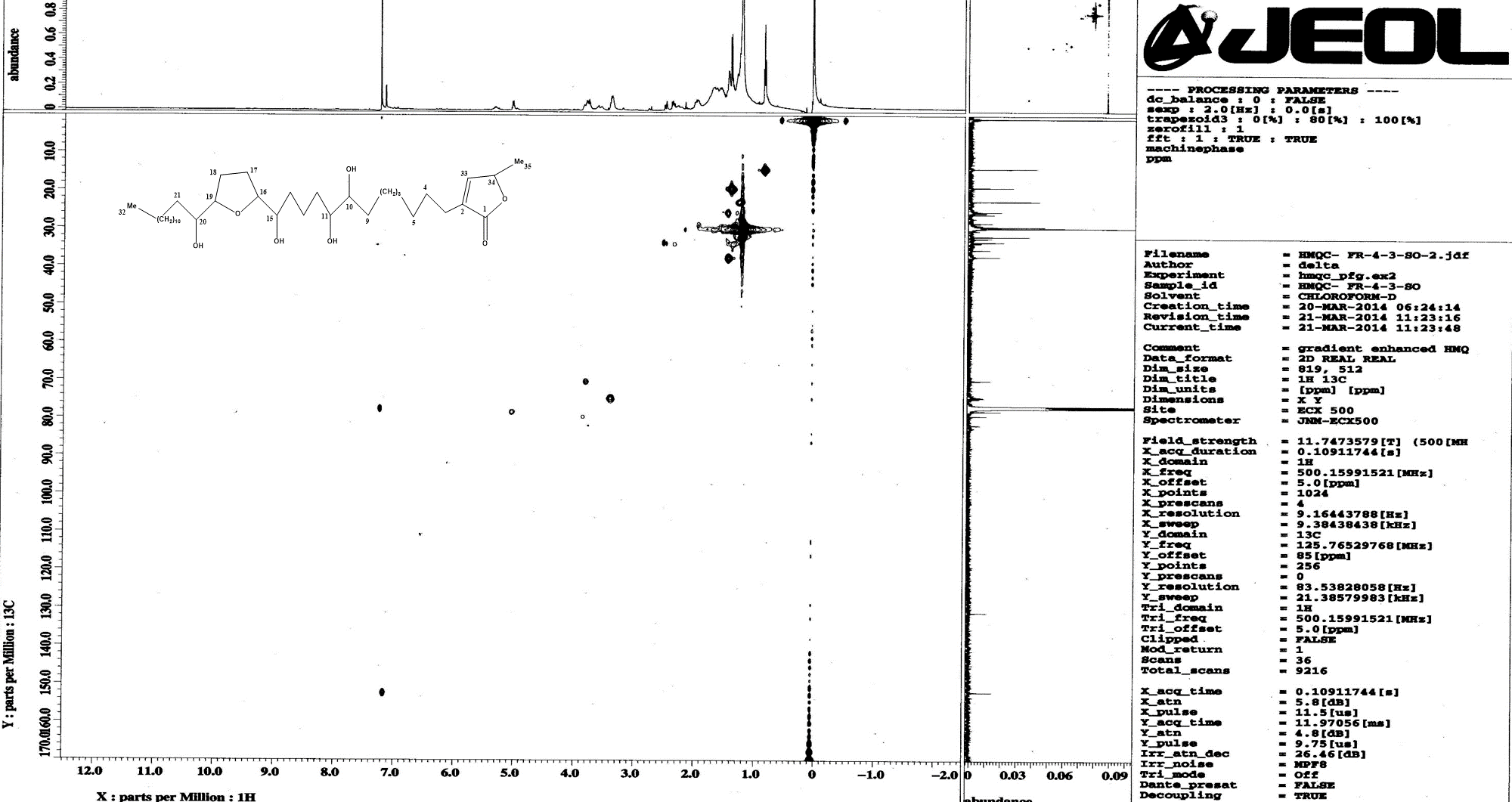


HMBC NMR of annomuricin E


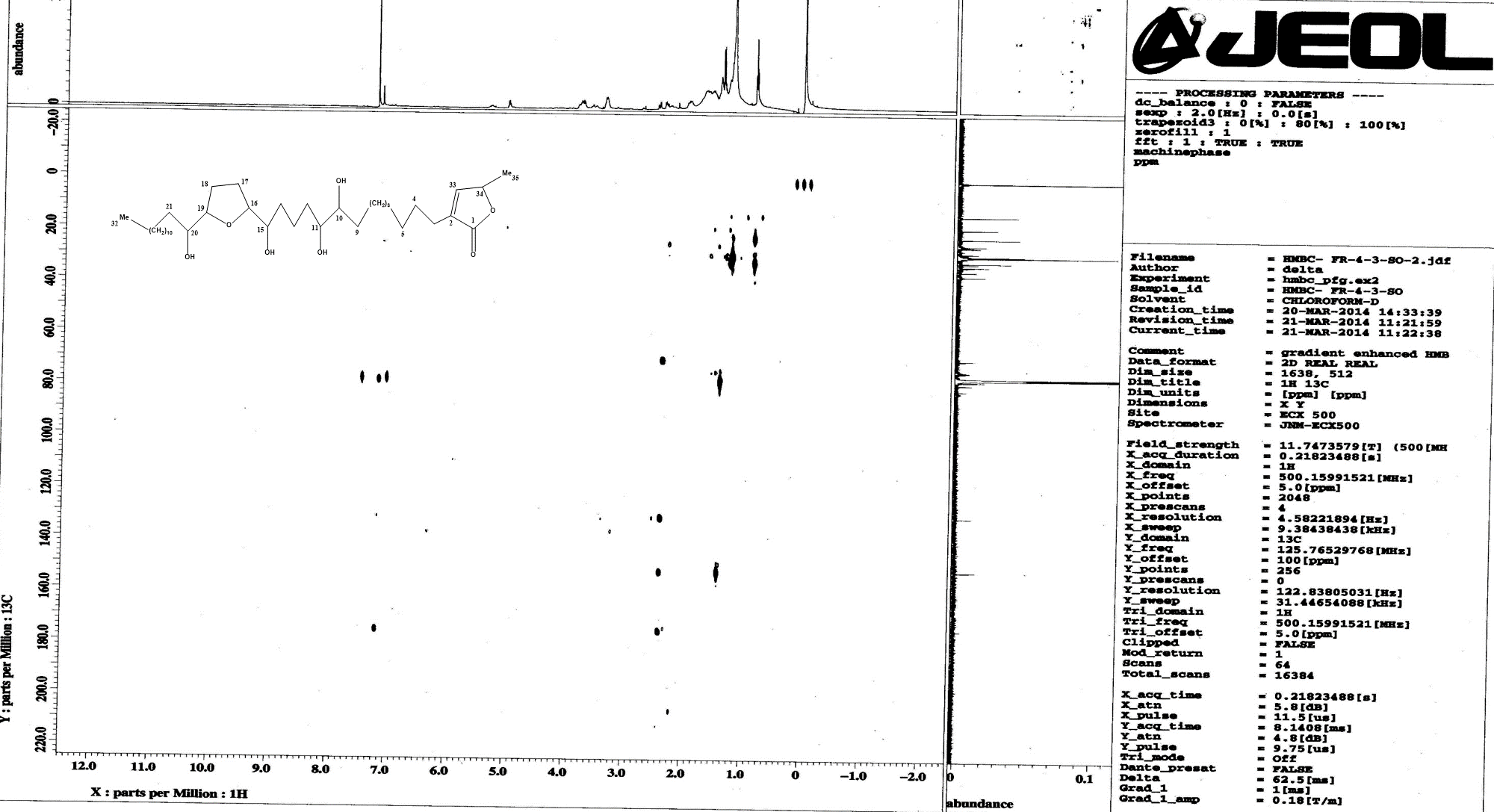


COSY NMR of annomuricin E


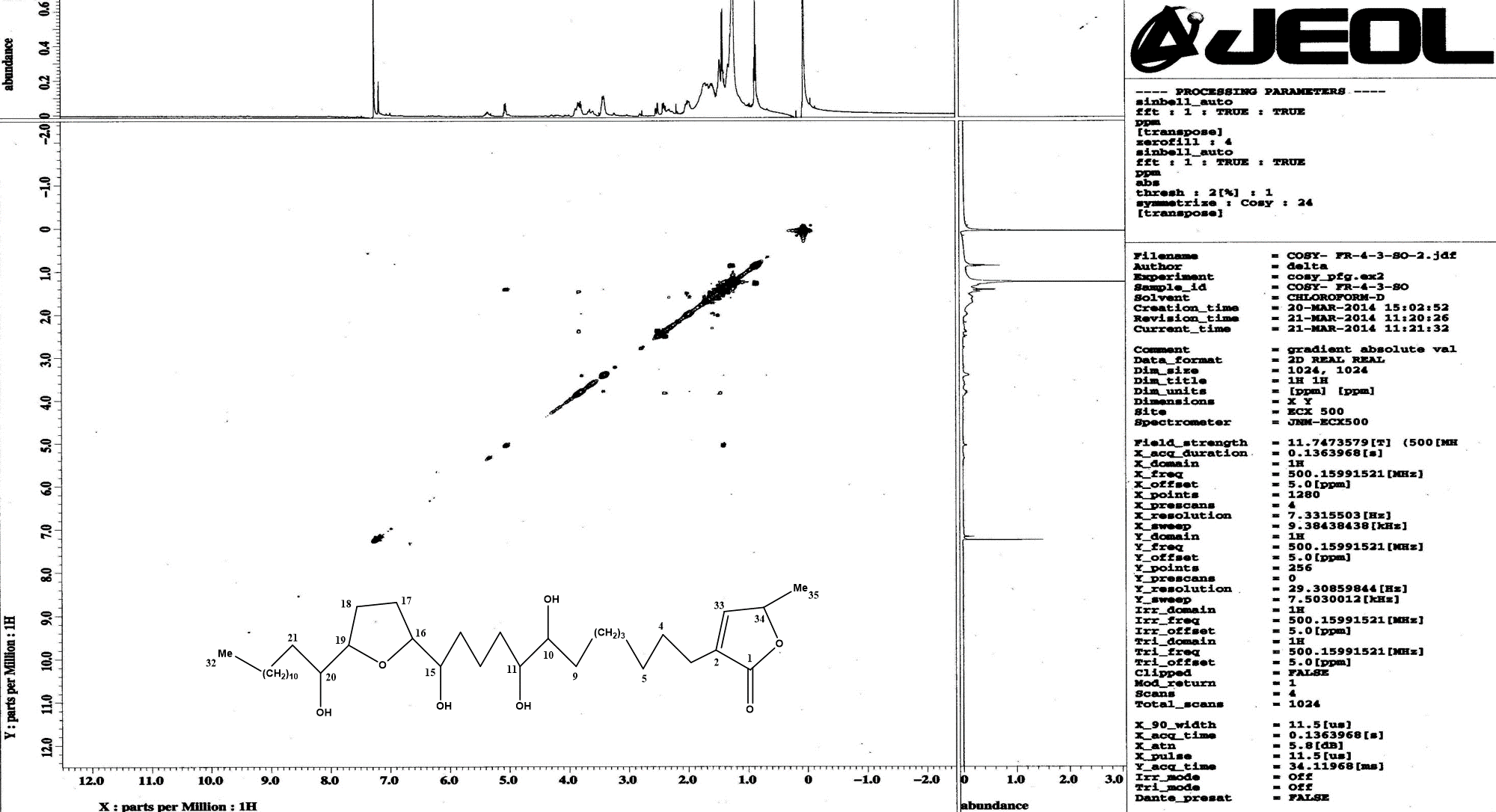


LC-MS of annomuricin E


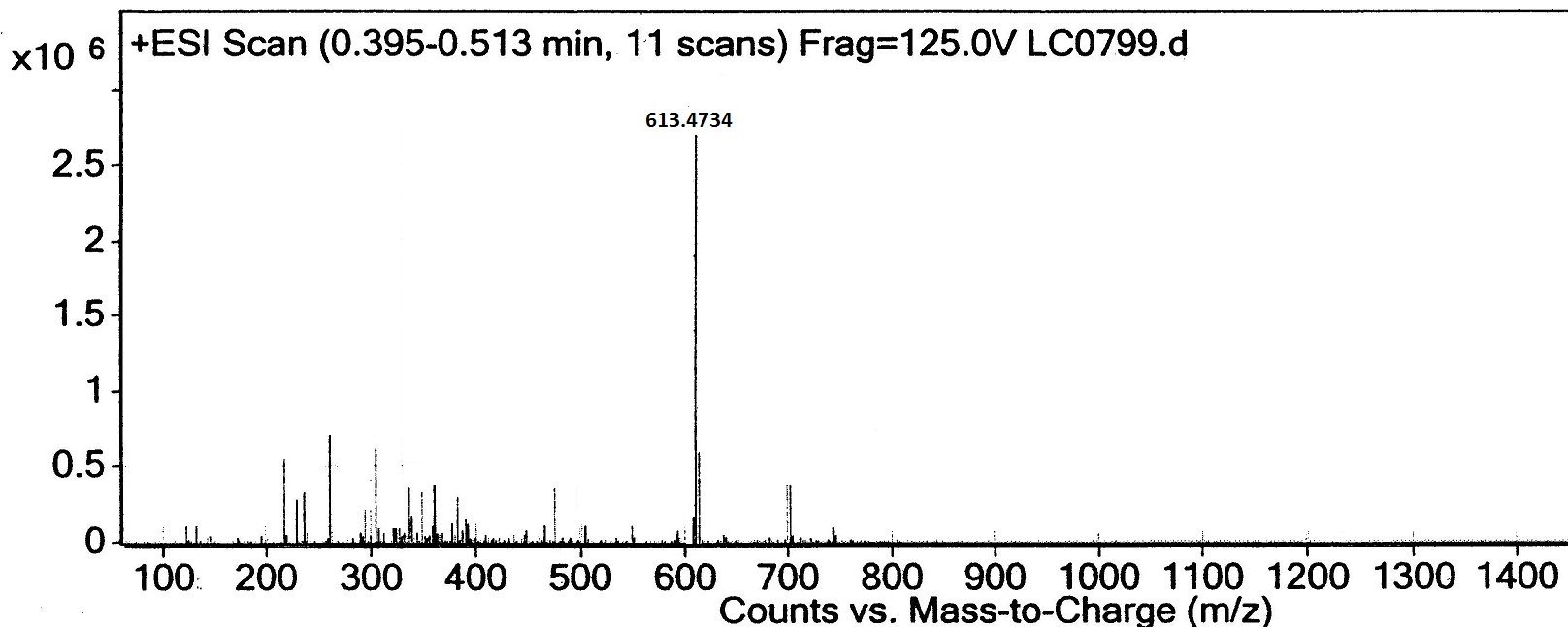

Supplement: S1 Fig — (DOCX) [file pone.0122288.s001.docx]
